# Comparison of the dipeptidyl peptidase-4 gene methylation levels between severely obese subjects with and without the metabolic syndrome

**DOI:** 10.1186/1758-5996-5-4

**Published:** 2013-02-04

**Authors:** Valérie Turcot, André Tchernof, Yves Deshaies, Louis Pérusse, Alexandre Bélisle, Picard Marceau, Frédéric-Simon Hould, Stéfane Lebel, Marie-Claude Vohl

**Affiliations:** 1 Institute of Nutraceuticals and Functional Foods (INAF), Pavillon des Services, Université Laval, 2440 Hochelaga Blvd, G1V 0A6, Québec, Canada; 2 Molecular Endocrinology and Genomics, CHUL Research Center, Québec, Canada; 3 Department of Food Sciences and Nutrition, Université Laval, Québec, Canada; 4 Department of Medicine, Université Laval, Québec, Canada; 5 Centre de Recherche de l’Institut Universitaire de Cardiologie et de Pneumologie de Québec, Québec, Canada; 6 Department of Social and Preventive Medicine, Université Laval, Québec, Canada; 7 Genotyping Platform Team, McGill University and Genome Quebec Innovation Center, Montréal, Canada; 8 Department of Surgery, Université Laval, Québec, Canada

**Keywords:** DNA methylation, Epigenetics, *DPP4* gene, Visceral adipose tissue, White blood cells, Plasma cholesterol

## Abstract

**Background:**

The dipeptidyl peptidase-4 (DPP4) enzyme is a novel adipokine potentially involved in the development of the metabolic syndrome (MetS). Previous observations demonstrated higher visceral adipose tissue (VAT) *DPP4* gene expression in non-diabetic severely obese men with (MetS+) vs. without (MetS−) MetS. *DPP4* mRNA abundance in VAT correlated also with CpG site methylation levels (%Meth) localized within and near its exon 2 (CpG_94_ to CpG_102_) in non-diabetic severely obese women, regardless of their MetS status. The actual study tested whether *DPP4 *%Meth levels in VAT are different between MetS− and MetS+ non-diabetic severely obese subjects, whether variable metabolic and plasma lipid profiles are observed between *DPP4 *%Meth quartiles, and whether correlation exists in *DPP4 *%Meth levels between VAT and white blood cells (WBCs).

**Methods:**

DNA was extracted from the VAT of 26 men (MetS−: n=12, MetS+: n=14) and 79 women (MetS−: n=60; MetS+: n=19), as well as from WBCs in a sub-sample of 17 women (MetS−: n=9; MetS+: n=8). The %Meth levels of CpG_94_ to CpG_102_ were assessed by pyrosequencing of sodium bisulfite-treated DNA. ANOVA analyses were used to compare the %Meth of CpGs between MetS− and MetS+ groups, and to compare the metabolic phenotype and plasma lipid levels between methylation quartiles. Pearson correlation coefficient analyses were computed to test the relationship between VAT and WBCs CpG_94-102_ %Meth levels.

**Results:**

No difference was observed in CpG_94-102 _%Meth levels between MetS− and MetS+ subjects in VAT (*P*=0.67), but individuals categorized into CpG_94-102_ %Meth quartiles had variable plasma total-cholesterol concentrations (*P*=0.04). The %Meth levels of four CpGs in VAT were significantly correlated with those observed in WBCs (r=0.55−0.59, *P*≤0.03).

**Conclusions:**

This study demonstrated that %Meth of CpGs localized within and near the exon 2 of the *DPP4* gene in VAT are not associated with MetS status. The actual study also revealed an association between the %Meth of this locus with plasma total-cholesterol in severe obesity, which suggests a link between the *DPP4* gene and plasma lipid levels.

## Background

Deposition of fat preferentially in the abdominal compartment is associated with metabolic and inflammatory alterations often referred to as the metabolic syndrome (MetS) which considerably increases the risk of type 2 diabetes and cardiovascular disease (CVD) [1]. Both unhealthy lifestyle and genetic predisposition may influence lipid storage and adipose tissue metabolism, as well as their underlying metabolic abnormalities [2]. In a search aimed at discovering novel candidate genes for MetS, a gene expression profiling of visceral adipose tissue (VAT) of non-diabetic severely obese men revealed ~1.85 fold higher expression of the dipeptidyl peptidase-4 (*DPP4*) gene in men MetS+ compared to MetS− [3,4].

The DPP4 (or CD26) glycoprotein is a transmembrane exoprotease expressed on epithelial cells, importantly in kidney, intestine and liver, as well as on endothelial cells, fibroblasts and lymphocytes (i.e. stimulated T-cells, B-cells and natural killer cells) [5-7]. Since DPP4 is known to inactivate incretin hormones involved in glucose-dependent insulin secretion, pharmacological DPP4-inhibitors have been developed to improve the treatment of type 2 diabetes [8]. Recently, Lamers *et al.* showed that DPP4 can be released from human differentiated adipocytes and can exert autocrine and paracrine effects by impairing insulin signalling [9]. The rate of DPP4 release from adipose tissue explants and soluble DPP4 plasma levels were both higher in severely obese subjects MetS+ as compared to subjects MetS−. The rate of DPP4 release was also significantly correlated with MetS phenotypes, such as waist circumference, insulin resistance, high-density lipoprotein (HDL)-cholesterol and triglycerides. The authors suggested that DPP4 may be a novel biomarker linking obesity to MetS [9]. It is thus meaningful to consider the *DPP4* gene as a good candidate for MetS development.

A detailed genetic investigation previously conducted at the *DPP4* locus in severely obese individuals revealed that *DPP4* single nucleotide polymorphisms were inconsistently associated with plasma triglycerides and total-cholesterol levels [4]. These polymorphisms were not significantly associated with *DPP4* mRNA abundance in VAT of non-diabetic severely obese women [4]. However, *DPP4* expression levels were negatively correlated with cytosine methylation levels of CpG sites (CpGs) within and near the second exon of the *DPP4* gene in non-diabetic severely obese women [10]. Percentage of methylation (%Meth) levels of the targeted CpGs were also positively correlated with plasma HDL-cholesterol levels in women [10]. In view of these observations, we hypothesized that the %Meth levels of CpGs within and near the second exon of the *DPP4* gene in VAT are associated with MetS susceptibility in non-diabetic severely obese subjects, which would then suggests its utility as a potential biomarker of MetS. We also hypothesized that *DPP4* methylation levels may be associated with plasma cholesterol levels, as opposed to *DPP4* polymorphisms [4], and that its methylation in peripheral white blood cells (WBCs) may serve as a surrogate measure of VAT methylation at the *DPP4* locus.

This study was thus undertaken to test whether the%Meth levels of the targeted CpGs at the *DPP4* locus in VAT are different between MetS− and MetS+ severely obese men and women and to verify whether the quartiles of %Meth are associated with metabolic and plasma lipid profiles. Correlations between *DPP4* %Meth levels in VAT and WBCs were tested in a sub-sample of severely obese women.

## Material and methods

### Patient selection

The study subjects were severely obese Caucasian men and women undergoing a biliopancreatic diversion with sleeve gastrectomy to treat obesity at the Centre de Recherche de l’Institut Universitaire de Cardiologie et de Pneumologie de Québec (Québec City, Québec, Canada) from June 2000 to June 2009. The diagnosis of type 2 diabetes mellitus was made prior to surgery [11]. Body weight, height, waist circumference, resting systolic and diastolic blood pressure were measured using standardized procedures [12]. The day of surgery, fasting blood samples were drawn into EDTA-containing tubes and centrifuged for WBCs DNA extraction and for plasma lipid and glucose concentration measurements [12]. VAT from the greater omentum was sampled during the surgery according to a standardized protocol, as previously reported [13,14]. All subjects provided a written informed consent to participate in this study which received the approval of the Université Laval Ethics Committee.

Non-diabetic subjects not using any medication to treat MetS components who were fulfilling minimal (MetS−: one to two MetS criteria) or maximal (MetS+: five MetS criteria) MetS criteria were selected. Based on the criteria from the International Diabetes Federation definition (IDF) [15], the selected MetS− subjects were centrally obese (waist circumference ≥88 cm for women and ≥102 cm for men; USA cutoff values [16]) with no more than one additional MetS criterion defined hereafter. On the opposite, the selected MetS+ subjects had to meet the five MetS criteria: waist circumference ≥88 cm for women and ≥102 cm for men, fasting plasma glucose ≥5.6 mmol/L, HDL-cholesterol <1.29 mmol/L for women and <1.03 mmol/L for men, triglycerides ≥1.7 mmol/L, systolic blood pressure (SBP) ≥130 mmHg or diastolic blood pressure (DBP) ≥85 mmHg. Postmenopausal women were excluded from this study in order to avoid potential confounding factors on *DPP4* gene expression and %Meth levels. Since there were fewer men meeting the five MetS criteria for the MetS+ group, the selection was extended to centrally obese men meeting at least three additional MetS criteria (i.e. total of four to five MetS criteria for men). To avoid significant differences between groups, MetS− vs. MetS+ men were matched for age, waist circumference and smoking. Thus, 28 men and 83 women were selected for this study. Considering the availability of VAT samples, we ended up with a total of 26 men (MetS−: n=12; MetS+: n=14) and 79 women (MetS−: n=60; MetS+: n=19) for the “VAT experiment”. Relative to the “WBCs experiment”, 28 non-smoking women were selected from the 79 women and matched for age and waist circumference between MetS− and MetS+ groups. Considering the availability of WBCs, we ended up with a sub-sample of 17 women (MetS−: n=9; MetS+: n=8) for whom VAT biopsies and WBCs were both available. Men were not considered for the “WBCs experiment” because of low WBCs availability.

### Analysis of *DPP4* CpG methylation in VAT and peripheral WBCs

**Figure 1:**
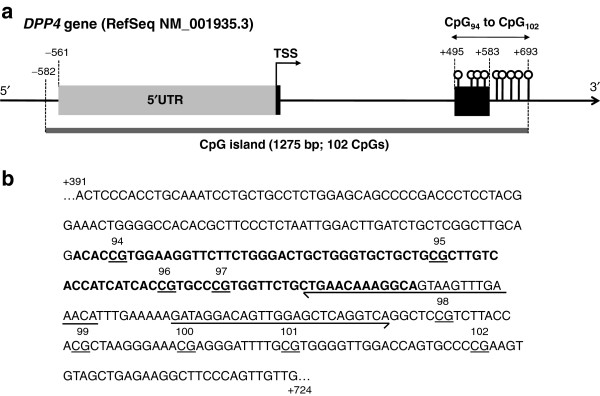
**Location of the 9 CpGs analyzed for their%Meth levels at the *DPP4 *locus. (a)** Delimitation of the 5^′^UTR, exons and CpG island at the *DPP4* locus were determined relatively to the first adenine (+1) from the ATG translation start site (TSS) based on the *DPP4* transcript NM_001935.3. The *DPP4* promoter CpG island was localized using the NCBI Map Viewer public database in a previous study [10]. **(b)** The DNA sequence displayed and surrounding the 9 CpG sites is located between the 391 and 724 bases after the TSS of the *DPP4* gene. The chromosomal location corresponds to chr2: 162929768-162930101 on the minus DNA strand as obtained using the Human Build 37.3 (http://www.ncbi.nlm.nih.gov/genome/guide/human/). Bold bases represent the exon 2 of the *DPP4* gene and arrows are the designed sequencing primers along with the sequencing direction. RefSeq, Reference Sequence.

DNA was extracted from VAT using the DNeasy Blood & Tissue kit (QIAGEN, Mississauga, Ontario, Canada) and from peripheral WBCs using the GenElute™ Blood Genomic DNA kit (Sigma, St Louis, MO, USA), as recommended by the manufacturers. DNA extracts were stored at −80ºC until quantitative methylation analysis using pyrosequencing technology from QIAGEN [17], which was performed by the McGill University and Genome Quebec Innovation Center (MUGQIC) Genotyping Platform team (Montréal, Québec, Canada). Treatment of DNA with sodium bisulfite, PCR amplifications and the pyrosequencing process were performed as previously described [10]. Primers designed by the MUGQIC Genotyping Platform team were used to amplify the end of the *DPP4* promoter CpG island (Figure 1a and 1b) which covered the nine CpGs that have previously been associated with *DPP4* gene expression in VAT as well as with plasma HDL-cholesterol levels in severely obese women [10]. The targeted region was amplified in two fragments using the following primer sequences: first fragment; forward, 5^′^-Biotin-AAT TGG GAA GTT TTT TTA GTT A-3^′^, reverse, 5^′^-TAC TAA ATA CTA CTA CCC TTA TCA CCA TCA TCA C-3^′^, sequencing, 5^′^-AAT AAA ACA ATT AAA ACT CAA ATC A; second fragment; forward, 5^′^-AAG AAG GAG TTT GAT TTG AGT TTT AAT TG-3^′^, reverse, 5^′^-Biotin-TCC CAC CTA CAA ATC CTA CTA CC-3^′^, sequencing, 5^′^- TGT TTT AAA TTT ATT GTT TTT GTT TAG-3^′^. PCR conditions may be obtained upon request. The mean%Meth for combined CpG_94_ to CpG_102_ (CpG_94-102_) in VAT and WBCs was calculated for each subject and used in the analyses along with individual CpG %Meth levels.

Additionally, *DPP4* mRNA abundance in VAT was measured in the study subjects. Total RNA was extracted from VAT to generate the cDNA [3] and used for the quantitative real-time reverse transcriptase polymerase chain reaction (qRT-PCR) analysis, as previously described [18]. Amplification efficiencies were validated and normalized to ribosomal 18S and quantities of target gene were calculated according to a standard curve. Primers and TaqMan probes overlapping exons 23 and 24 of the *DPP4* gene (transcript NM_001935) and those for the ribosomal 18S were obtained from Applied Biosystems (*DPP4*: Hs00897398_m1; r18S: Hs99999901_s1). Normalized *DPP4* gene expression levels were available for 104 subjects (one excluded outlier; a MetS− woman).

### Statistical analysis

Non-normally distributed phenotypes, %Meth levels of individual CpGs and CpG_94-102_, as well as *DPP4* mRNA abundance in the 105 subjects selected for the “VAT experiment” were log_10_- or negative inverse-transformed. The normality of the distribution of all these variables was also verified independently in the sub-sample of 17 women selected for the “WBCs experiment” and they were transformed when needed. The general linear model (GLM) and the type III sum of squares in SAS were used to perform ANOVA analyses to compare the phenotype levels, the %Meth of individual CpGs and CpG_94-102,_ and *DPP4* mRNA abundance between MetS− and MetS+ groups while including sex in the model (adjustment for the sex effect). Associations between *DPP4* %Meth levels and metabolic profile, as well as with plasma lipid levels were tested by dividing the study subjects into quartiles based on their CpG_94-102_ %Meth levels and by comparing mean phenotype levels between quartiles using ANOVA analyses as described above while including potential confounding factors in the model (see the Results section for more details about adjustments). When a significant effect of CpG_94-102_%Meth quartiles on phenotypes was noted, least square means were used to identify quartiles which are significantly discordant in the corresponding phenotype. For the MetS status, logistic regression analysis was performed to compare MetS− and MetS+ frequencies between CpG_94-102_ %Meth quartiles. Finally, a Pearson correlation coefficient was computed to test the relationship between VAT and WBCs CpG_94-102_ %Meth levels in the sub-sample of 17 women selected for the “WBCs experiment”. The statistically significant *P*-value was set at 0.05. Statistical analyses were performed using SAS software version 9.2 (SAS Institute, Cary, NC).

## Results

### Characteristics of the study subjects in MetS− and MetS+ groups

The characteristics of the subjects are presented in Table 1 for each type of experiment (VAT and WBCs). As expected, no significant difference was observed regarding smoking habit, age and anthropometric values between MetS groups, as well as with total- and low-density lipoprotein (LDL)-cholesterol levels. In contrast and by design, all other MetS-related phenotypes (i.e. fasting glucose, triglycerides, HDL-cholesterol, SBP and DBP) differed between MetS− and MetS+ groups when sex was included in the model (*P* < 0.01), except for SBP level in the “WBCs experiment” which was not significantly different between MetS groups (*P* = 0.34).

**Table 1:** **Characteristics of the subjects for *DPP4*%Meth analysis in VAT and WBCs**

	**VAT experiment**		**WBCs experiment**	
**Phenotypes**	**MetS−**	**MetS+**	**MetS−**	**MetS+**
Number of men/women (n)	12/60	14/19	0/9	0/8
Number of MetS criteria (n)	1-2	4-5	1-2	5
Smokers (n)	13 (18.1%)	5 (16.1%)	0	0
Age (years)	35.3±8.8	35.4±7.5	35.6±8.4	36.1±8.5
BMI (kg/m^2^)	49.7±8.4	53.6±13.2	47.6±5.6	47.4±5.4
Waist circumference (cm)	131.3±18.7	145.0±22.0	132.3±10.5	125.6±10.3
Fasting glucose (mmol/L)	4.87±0.36^**^	6.23±0.71^**^	4.90±0.38^**^	6.05±0.43^**^
Triglycerides (mmol/L)	1.12±0.32^**^	2.73±1.50^**^	1.19±0.34^**^	3.19±2.14^**^
HDL-cholesterol (mmol/L)	1.50±0.27^**^	1.02±0.16^**^	1.48±0.30^**^	0.99±0.19^**^
LDL-cholesterol (mmol/L)	2.80±0.77	3.03±0.90	2.70±0.64	2.74±1.06
Total-cholesterol (mmol/L)	4.81±0.84	5.17±0.89	4.72±0.72	4.93±1.03
SBP (mmHg)	127.3±13.2^**^	149.5±15.6^**^	138.8±17.0	145.4±9.0
DBP (mmHg)	76.9±6.0^**^	93.1±9.6^**^	78.9±5.8^**^	95.9±6.7^**^

Mean±SD or n are presented in the table.

All the study subjects were non-diabetic and were not using any medication to treat MetS components.

Non-normally distributed phenotypes were transformed for the comparison of mean phenotype levels between MetS groups; for the VAT experiment: body mass index (BMI) (−1/(X)), fasting glucose (−1/(X)), triglycerides (−1/(1+X)) and DBP (−1/(X)); for the WBCs experiment: triglycerides (log_10_X) and HDL-cholesterol (log_10_X). ^**^P < 0.01.

### Comparison of *DPP4* %Meth levels between MetS− and MetS+ groups

**Figure 2:**
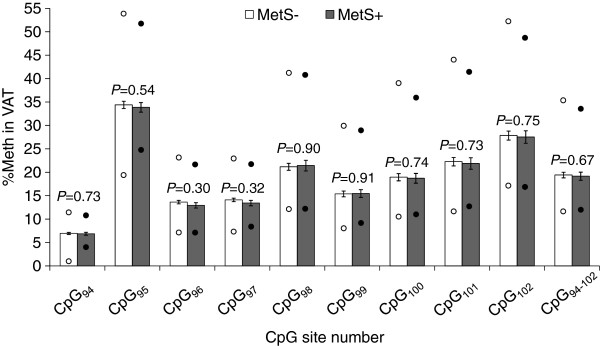
**VAT methylation levels and their comparison between MetS− and MetS+ groups in severely obese subjects.** Non-normally distributed%Meth levels were transformed for the analysis using the negative inverse (-1/X) for CpG_98_, CpG_99_, CpG_100_, CpG_101_, CpG_102_ and CpG_95-102_. Mean±s.e.m. are presented in the figure. MetS−: n=72 subjects; MetS+: n=33 subjects. Lower circles: minimum%Meth value; Upper circles: maximum%Meth value. NT, not-tested; s.e.m., standard error of mean.

**Figure 3:**
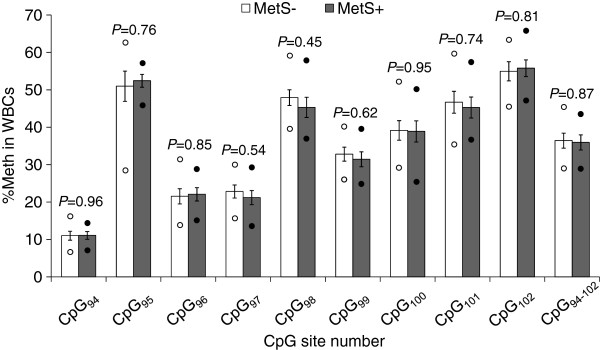
**WBCs methylation levels and their comparison between MetS− and MetS+ groups in severely obese women.** Mean±s.e.m. are presented in the figure. Lower circle: minimum %Meth value; Upper circle: maximum %Meth value. MetS−: n=9 women; MetS+: n=8 women. NT, not-tested; s.e.m., standard error of mean.

The methylation analysis of the nine targeted CpGs revealed that their %Meth levels were highly correlated between each other in VAT (r = 0.71−0.99; *P* < 0.0001), as well as in WBCs (r = 0.70−0.98; *P* < 0.0001). Mean %Meth levels of individual CpGs and CpG_94-102_ in VAT were similar in MetS− and MetS+ subjects when sex was included in the model (*P* ≥ 0.30) (Figure 2). Similar results were observed in sex-specific analyses (Men: *P* ≥ 0.62; Women: *P* ≥ 0.18; data not shown). Further, *DPP4* mRNA abundance analysis revealed that the *DPP4* gene was equally expressed (log_10_-transformed) in the VAT of MetS− and MetS+ subjects in the present study when sex was included in the model (*P* = 0.21), which was also the case in sex-specific analyses (Men: *P* = 0.64; Women: *P* = 0.06; data not shown). Along with these results, no difference was observed in the %Meth levels of individual CpGs and CpG_94-102_ in WBCs between MetS− and MetS+ subjects (*P* ≥ 0.45) (Figure 3).

### Associations between *DPP4* %Meth quartiles in VAT and the metabolic and lipid profiles

**Figure 4:**
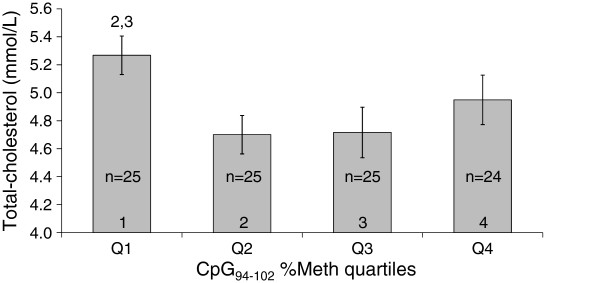
**Mean total-cholesterol concentrations between CpG**_**94-102 **_**%Meth quartiles in VAT of severely obese subjects.** Mean±s.e.m. are presented in the figure.

The study subjects were divided into quartiles (Q) based on their CpG_94-102_ %Meth levels in VAT (Q1: 11.75%−15.74%; Q2: 15.74%−18.02%; Q3: 18.02%−21.25%; Q4: 21.25%−35.47%; n = 26 in each quartile). As observed in Table 2, frequencies of MetS− and MetS+ subjects and mean MetS phenotype levels were similar between CpG_94-102_ %Meth quartiles, except for a tendency towards differences in plasma triglyceride levels between CpG_94-102_ %Meth quartiles (unadjusted *P* = 0.07; adjusted *P* = 0.08; see legend of Table 2 for details about adjustments). Regarding plasma lipid levels, a significant association was observed between CpG_94-102_ %Meth quartiles and total-cholesterol concentrations (unadjusted *P* = 0.03; adjusted *P* = 0.04), while no association was found between %Meth quartiles and mRNA abundance (unadjusted *P* = 0.13; adjusted *P* = 0.13). As observed in Figure 4, subjects with the lowest CpG_94-102_ %Meth quartile (Q1) had higher total-cholesterol concentrations as compared to subjects in the second (Q2; *P* = 0.01) and the third (Q3; *P* = 0.02) CpG_94-102_ %Meth quartiles when including age, sex, smoking and waist circumference in the model. Additional CpG site-specific analyses revealed that total-cholesterol and triglyceride levels were significantly different between CpG_98_ (*P* = 0.03) and CpG_99_ (*P* = 0.005) %Meth quartiles, respectively, when including age, sex, smoking and waist circumference in the model. Globally, a u-shape relationship was observed where CpG_98_ %Meth Q3 had lower plasma total-cholesterol levels as compared to Q1 (*P* = 0.007) and Q4 (*P* = 0.02), and where CpG_99_ %Meth Q2 had lower plasma triglycerides as compared to Q1 (*P* = 0.008), Q3 (*P* = 0.0009) and Q4 (*P* = 0.01) (data not shown).

**Table 2:** **Comparison of study subjects’ characteristics between quartiles of CpG**_**94-102**_**%Meth levels measured in VAT**

**CpG**_**94-102 **_**%Meth quartiles**	**Q1 [11.75−15.74[**	**Q2 [15.74−18.02[**	**Q3 [18.02−21.25[**	**Q4 [21.25−35.47]**	***P***_**quartiles**_	***P***_**quartiles**_
	**n = 26**	**n = 26**	**n = 26**	**n = 26**	**Unadjusted**	**Adjusted**
***MetS phenotypes***						
MetS− (n = 72)	16 (0.22)	20 (0.28)	19 (0.26)	17 (0.24)	−	−
MetS+ (n = 32)	10 (0.31)	6 (0.19)	7 (0.22)	9 (0.28)	0.62	0.53
Waist circumference (cm)	135.6 ± 4.2	135.8 ± 4.2	133.3 ± 4.5	138.1 ± 3.7	0.87	0.78
Fasting glucose (mmol/L)	5.36 ± 0.13	5.08 ± 0.11	5.26 ± 0.17	5.44 ± 0.20	0.48	0.57
Triglycerides (mmol/L)	1.73 ± 0.15	1.24 ± 0.10	1.61 ± 0.30	1.91 ± 0.29	0.07	0.08
HDL-cholesterol (mmol/L)	1.37 ± 0.06	1.41 ± 0.06	1.37 ± 0.07	1.26 ± 0.06	0.42	0.62
SBP (mm Hg)	135.7 ± 3.0	138.0 ± 3.7	131.7 ± 3.34	131.1 ± 3.5	0.42	0.23
DBP (mm Hg)	83.0 ± 1.6	81.0 ± 2.3	81.9 ± 1.9	81.5 ± 2.3	0.78	0.56
***Other phenotypes***						
LDL-cholesterol (mmol/L)	3.10 ± 0.13	2.68 ± 0.14	2.75 ± 0.17	3.00 ± 0.19	0.19	0.26
Total-cholesterol (mmol/L)	5.26 ± 0.13	4.65 ± 0.14	4.72 ± 0.18	5.10 ± 0.19	**0.03**	**0.04**
*DPP4* mRNA in VAT (ratio)	3.92 ± 0.98	5.37 ± 1.24	2.99 ± 0.92	3.29 ± 0.81	0.13	0.13

*P*-values shown are those obtained for the effect of%Meth quartiles when unadjusted or adjusted for potential confounding factors (age, sex and smoking for MetS and waist circumference; age, sex, smoking and waist circumference for the other phenotypes). Log10-transformed for *DPP4* mRNA abundance and negative inverse-transformed for fasting glucose, triglycerides and DBP were used in the analyses. Mean ± s.e.m. or n are shown in the table.

### Correlations between VAT and peripheral WBCs *DPP4*%Meth levels

In the premenopausal severely obese women selected for the “WBCs experiment”, CpG_94-102_ %Meth levels in VAT tended to be correlated with their %Meth levels in WBCs (r = 0.43, *P* = 0.09). Regarding CpG site-specific analyses,%Meth levels of four CpGs in VAT, CpG_95_ (r = 0.59, *P* = 0.01)_,_ CpG_99_ (r = 0.55, *P* = 0.03)_,_ CpG_100_ (r = 0.55, *P* = 0.03) and CpG_101_ (r = 0.56, *P* = 0.02), correlated significantly with their %Meth in WBCs.

## Discussion

This study revealed that the mean%Meth levels of CpGs located within and near the second exon of the *DPP4* gene (CpG_94_ to CpG_102_) were comparable in VAT of non-diabetic severely obese MetS− and MetS+. Subjects classified into quartiles based on their CpG_94-102_ %Meth levels had similar characteristics regarding MetS phenotypes, but were discordant for their plasma total-cholesterol levels. In a sub-sample of non-diabetic severely obese premenopausal women, CpG_94-102_ %Meth levels in VAT tended to be correlated with their %Meth levels in WBCs, and %Meth levels of four individual CpGs (CpG_95_, CpG_99_, CpG_100_ and CpG_101_) were significantly correlated.

The presence of similar *DPP4* %Meth levels in VAT between MetS− and MetS+ subjects rejects the previous hypothesis of a significant difference between the two groups [3,4]. This observation may potentially be explained by the absence of differential *DPP4* gene expression in VAT of the studied men and women, which contrasts with the differential expression observed in our previous studies on severely obese men MetS− (n=7) and MetS+ (n=7) [3,4]. It is actually difficult to clearly explain this discrepancy. Beside sex, the same subject selection criteria were applied and the characteristics of male subjects were quite similar as those from the microarray study [3]. Potential predictors of *DPP4* gene expression in VAT were derived from a complimentary stepwise regression analysis (predictive variables: age, sex, waist circumference and smoking; data not shown). Only smoking was significantly associated with *DPP4* mRNA levels in combined men and women (r^2^=0.05, *p*=0.02). The proportion of smokers among the study subjects was lower as compared to the microarray experiment where about half of subjects in each group were smokers [3]. Since frequencies of smokers were not different between MetS groups in the actual and the microarray studies, it would thus potentially anneal the effect of smoking when performing *DPP4* mRNA comparisons between MetS groups. However, it may not reveal potential interactions with smoking which would have induced a greater *DPP4* expression among MetS+ men taking part of the microarray experiment as compared to male subjects herein. However, to the best of our knowledge, no published studies reported that smoking was related with differential *DPP4* gene expression. Added to this explanation, other subjects’ characteristics which were not evaluated in our present and previous studies may have confounded *DPP4* gene expression, such as other *DPP4* locus regions under epigenetic regulation [19,20] via stimulation by cytokines [21,22], the type of cells (e.g. adipocytes vs. stromo-vascular cells) and their differentiation state [7] within VAT, or even the adipocyte volume [9]. Further epigenetic and expression studies of the *DPP4* gene in cell-specific analyses and with some VAT structural characterization may potentially help to better understand the previous *DPP4* differential expression observed in VAT of non-diabetic severely obese men MetS− and MetS+ [3,4].

This study aimed also to test whether the metabolic and plasma lipid profiles are variable between *DPP4*%Meth quartiles in VAT. Apart from different plasma triglyceride concentrations between CpG_99_ %Meth quartiles, similar MetS phenotypes were observed between individual CpGs and combined CpG_94-102_ %Meth quartiles. In a previous study, positive correlation between plasma HDL-cholesterol levels and CpG_94-102_ %Meth levels in VAT of non-diabetic severely obese women were observed [10]. However, the actual study did not reveal any association between HDL-cholesterol concentrations and CpG_94-102_ %Meth levels (Pearson correlation analysis; data not shown) or CpG_94-102_ %Meth quartiles, even in sex-specific analyses (data not shown). Even though the actual and the previous studies [10] revealed discordant associations between *DPP4* %Meth in VAT and MetS phenotypes, they both underlined an association between the *DPP4* gene and plasma lipid profile. This observation is further supported by the presence of significant differences in total-cholesterol concentrations between CpG_94-102_ %Meth quartiles in this study, which are greater in subjects within Q1 as compared to those within Q2 and Q3, but without any difference between quartiles in *DPP4* mRNA abundance in VAT. A previous genetic investigation at the *DPP4* locus also revealed that two common single nucleotide polymorphisms were inconsistently associated with the risk of high triglyceride and cholesterol levels in a multi-stage study design conducted in severely obese individuals [4]. These results suggest that both *DPP4* %Meth levels (in VAT) and polymorphisms may influence the association between the *DPP4* gene and the plasma lipid profile, such as triglycerides and total-cholesterol levels. This hypothesis may partly explain the inconsistency seen in the relation between *DPP4* genetic and epigenetic variations with the plasma lipid profile and with *DPP4* mRNA levels in VAT. Larger studies which would include both types of variations may increase the chance to observe genetic associations in future studies. Their functional impacts on *DPP4* gene expression and function (protein and activity), as well as on DPP4-cleaved protein levels, would then merit further investigations.

The link between DPP4 and the lipid profile contrasts with its well-known role on glucose homeostasis. DPP4 has recently been identified in VAT [9] and its function within this tissue is unknown. A potential link with plasma triglycerides could be made by the assumption that DPP4 may inhibits the effect of the incretin hormone glucose-dependent insulinotropic polypeptide on lipoprotein lipase synthesis and activity in adipocytes [23,24], which would favor hypertriglyceridemia due to lower triglyceride hydrolysis as seen in lipoprotein lipase deficiency [25]. The link between DPP4 in VAT and total-cholesterol concentrations is somewhat more difficult to delineate, but some assumptions could be made when considering DPP4 action within other tissues and plasma. Inhibition of DPP4 with vitaglipin has shown to increase the active form of glucagon-like peptide-1 along with a reduction in cholesterol content of chylomicrons after a fat-rich diet in type 2 diabetic subjects [26], which suggests an indirect link between DPP4 function and cholesterol absorption. Tahara *et al.* have also recently observed a dose response relationship between DPP4 protein levels and plasma total-cholesterol concentrations. However, in a multiple stepwise regression analysis HDL-cholesterol, but not total-cholesterol, was independently associated with DPP4 plasma levels in a Japanese population [27]. DPP4 is also recognized as a potential peptidase involved in the truncation of the neuropeptide Y (NPY), which would modulate its receptor preference [6]. The common SNP Leu(7)-to-Pro(7) (T1128C) in the *NPY* gene has previously been associated with higher synthesis and secretion of NPY [28], and also with higher total- and LDL-cholesterol concentrations in serum of obese Finnish and Dutch subjects [29]. It is unknown how NPY may be related with serum total-cholesterol concentrations in this study [29], but these results suggest that an indirect link between DPP4 and total-cholesterol may exist via the modulation of NPY function. Furthermore, a recent study conducted by Zhang *et al.* also demonstrated an association between *DPP4* expression and protein levels of two enzymes involved in cholesterol biosynthesis in melanoma cells [30], but it is unknown if this relation could be observed in other tissues, such as in the liver. Hence, these observations demonstrate few evidences relating the DPP4 enzyme with the lipid profile, which may be further clarified in other independent studies.

Currently, there is concern as to whether less-invasively obtainable human samples (i.e. blood and saliva) would be good surrogates for disease-associated epigenetic biomarkers, such concerns being raised because of the tissue specificity of both epigenetic patterns [31,32] and disease-associated epigenetic variations [33]. In the actual study, correlations were observed between the %Meth levels of some targeted CpGs in VAT and WBCs of a sub-sample of severely obese women. Although cell types expressing *DPP4* mRNA in VAT and WBCs were not investigated in this study, lymphocytes are suspected to be a common link between the two compartments because they are DPP4-expressing cells (i.e. stimulated T-cells, B-cells and natural killer cells) [5-7] and they are present in both obese VAT [34-36] and peripheral WBCs. This hypothesis may thus partly explain the relationship seen between WBCs and VAT %Meth levels.

One study limitation needs to be outlined, which regards the heterogeneity of MetS definition to categorize subjects as being affected or not by obesity-related metabolic complications. To overcome this issue, the selection of non-diabetic individuals in the extremes of the MetS definition has been attempted in this study. However, it may not take into account other metabolic (e.g. insulin resistance) and VAT physiological (e.g. adipocyte size, immune cell infiltration) parameters that may better discriminate those expressing *DPP4* at greater levels in their VAT [3,4]. As previously underlined, further epigenetic and expression studies at the *DPP4* locus would be needed to clarify what controls VAT *DPP4* expression and whether its methylation levels influence gene expression in a cell-specific fashion.

In conclusion, this study demonstrated that %Meth of CpGs localized within and near the exon 2 of the *DPP4* gene in VAT are not associated with MetS status. The actual study also revealed an association between the *DPP4* %Meth with plasma total-cholesterol levels in severe obesity, which suggests a link between the *DPP4* gene and plasma lipid metabolism. Finally, since the %Meth levels of some of the targeted *DPP4* CpGs in VAT correlated with those observed in WBCs, peripheral WBCs may potentially be used as a surrogate measure for *DPP4* methylation analysis in further epidemiological studies in relation with the plasma lipid profile.

## Abbreviations

CpG: Cytosine-phosphate-guanine; CVD: Cardiovascular disease; DPP4: Dipeptidyl peptidase-4; GLM: General linear model; HDL: High-density lipoprotein; IDF: International Diabetes Federation; LDL: Low-density lipoprotein; MetS: Metabolic syndrome; MetS−: without metabolic syndrome; MetS+: with metabolic syndrome; NPY: Neuropeptide Y; %Meth: percentage of methylation; qRT-PCR: quantitative real-time reverse transcriptase polymerase chain reaction; SBP: Systolic blood pressure;DBP: Diastolic blood pressure; VAT: Visceral adipose tissue; WBCs: White blood cells

## Competing interests

The authors declare that they have no competing interests.

## Authors’ contributions

VT participated to the study design, performed the statistical analyses, interpreted the data and drafted the manuscript. AT, YD, and LP participated to the elaboration of the study design. AB was in charge of the methylation analysis. PM, FSH and SL sampled blood and adipose tissue from the study subjects. MCV conceived and designed the study. All authors read and approved the final manuscript.
